# A multilayer perceptron-based model applied to histopathology image classification of lung adenocarcinoma subtypes

**DOI:** 10.3389/fonc.2023.1172234

**Published:** 2023-05-18

**Authors:** Mingyang Liu, Liyuan Li, Haoran Wang, Xinyu Guo, Yunpeng Liu, Yuguang Li, Kaiwen Song, Yanbin Shao, Fei Wu, Junjie Zhang, Nao Sun, Tianyu Zhang, Lan Luan

**Affiliations:** ^1^ Key Laboratory of Geophysical Exploration Equipment, Ministry of Education, College of Instrumentation and Electrical Engineering, Jilin University, Changchun, China; ^2^ Department of Thoracic Surgery, The First Hospital of Jilin University, Changchun, China; ^3^ Department of Pathology, Central Hospital Affiliated to Shenyang Medical College, Shenyang, China; ^4^ Center for Reproductive Medicine and Center for Prenatal Diagnosis, The First Hospital of Jilin University, Changchun, China

**Keywords:** histopathology image, lung cancer, deep learning, multilayer perceptron, image classification

## Abstract

**Objective:**

Lung cancer is one of the most common malignant tumors in humans. Adenocarcinoma of the lung is another of the most common types of lung cancer. In clinical medicine, physicians rely on the information provided by pathology tests as an important reference for the fifinal diagnosis of many diseases. Thus, pathological diagnosis is known as the gold standard for disease diagnosis. However, the complexity of the information contained in pathology images and the increase in the number of patients far exceeds the number of pathologists, especially in the treatment of lung cancer in less-developed countries.

**Methods:**

This paper proposes a multilayer perceptron model for lung cancer histopathology image detection, which enables the automatic detection of the degree of lung adenocarcinoma infifiltration. For the large amount of local information present in lung cancer histopathology images, MLP IN MLP (MIM) uses a dual data stream input method to achieve a modeling approach that combines global and local information to improve the classifification performance of the model. In our experiments, we collected 780 lung cancer histopathological images and prepared a lung histopathology image dataset to verify the effectiveness of MIM.

**Results:**

The MIM achieves a diagnostic accuracy of 95.31% and has a precision, sensitivity, specificity and F1-score of 95.31%, 93.09%, 93.10%, 96.43% and 93.10% respectively, outperforming the diagnostic results of the common network model. In addition, a number of series of extension experiments demonstrated the scalability and stability of the MIM.

**Conclusions:**

In summary, MIM has high classifification performance and substantial potential in lung cancer detection tasks.

## 1 Introduction

Lung cancer has become one of the major cancer diseases in the world (1). Breast cancer and prostate cancer are more common among women and men, respectively, but the incidence and mortality rates of both cancers are much lower than those of lung cancer. Lung adenocarcinoma is the most common type of lung cancer, accounting for about 50% of lung cancers (2). Microinvasive lung adenocarcinoma is an early type of lung cancer that is detected in a timely manner whose tumor cells can be completely removed through radical surgery (3). Invasive lung adenocarcinoma is relatively more malignant and more complicated to treat than noninvasive ones. Timely and accurate determination of the type of lung cancer can greatly improve the cure rate of patients.

The most reliable scientific basis for physicians to diagnose lung cancer is through pathology (4). Morphological observation of tumor cells under a microscope revealed that different types of tumor cells have different nuclei, cell size, and morphology (5). However, sometimes the microscope cannot clearly identify tumor type; hence, tumor type has to be further determined by the indexes obtained by immunohistochemistry. If the tumor is small, then immunohistochemistry cannot also give a good judgment. Current treatment methods for lung cancer are limited, irreversible, and harmful to the human body. Therefore, accurate diagnosis prior to lung cancer treatment is necessary to avoid unnecessary harm to patients.

Deep learning has become widely used in different fields with the rapid development of artificial intelligence (6). and increasing studies are showing the reliability of deep learning algorithms in medical image analysis (7). Some examples are as follows: Hua et al. (8) applied the DBN to classify lung nodules as malignant or benign with a sensitivity rate of 73.40% and a specificity rate of 82.20%.Roy et al. (9) studied a system based on fuzzy inference system for the image classification of cancerous and non-cancerous lung tumors. In a paper published in 2018 at New York University School of Medicine (10), a deep learning-based triple classification task for non-small cell lung cancer histopathology slices was explored, and verified the reliability of CNNs applied to pathology tasks. In 2019, X. Wang et al. (11) proposed a new method based on weakly supervised learning to solve the classification problem of pathological sections of lung cancer. W. Shen et al. (12) designed an end-to-end deep learning architecture, the multi-crop CNN, for low- and high-malignancy lung nodule classification. Kriegsmann et al. (13) used a CNN-based model for the classification of common lung cancer subtypes. The study highlights the potential of CNN image classification models for tumor differentiation. Taken together, this demonstrates that deep learning tools are feasible for some aspects of renal pathology classification. Khademi et al. (14) proposed a novel spatio-temporal fusion model using convolutional autoencoder to extract features from CT scans and Swin Transformer to process the time series features of clinical data. The model shows high accuracy and stability in the malignancy prediction of lung cancer.

Convolutional neural networks (CNNs) (15) and vision transformers (VTs) (16) have emerged as popular deep learning models for various computer vision tasks, including image segmentation and classification (17). While CNNs and VTs have shown promising results, they often require complex network architectures and high computational costs. To address these challenges, a new model called MLP-Mixer (18) has been proposed by the Google team. Unlike CNNs and VTs, MLP-Mixer relies entirely on multilayer perceptron (MLP) and does not use any convolutional operations or self-attentive mechanisms, resulting in a simpler network architecture and lower computational requirements. Therefore, MLP-Mixer offers a promising alternative for efficient and effective image processing tasks. MLP-Mixer only applies MLP to process the global information between patch sequences to achieve classification tasks, which can obtain a performance comparable to those of CNN and VT. The morphology and volume of cell nuclei in histopathological images are different; therefore, these images have a large amount of local information. Moreover, MLP-Mixer destroys the local structure of patches and cannot capture local information; hence, the application of MLP-Mixer model to histopathological images has a shortage.

**Figure 1:**
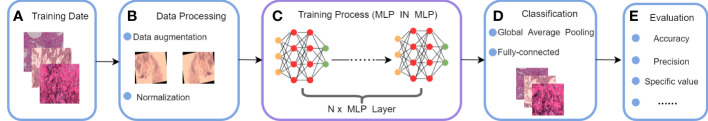
Workflow of the proposed MLP IN MLP model: **(A)** train date, **(B)** date process, **(C)** train process, **(D)** output classification results, **(E)** Assessing performance.

The MLP IN MLP (MIM) proposed in this study is also architected as a MLP, but it notices the information features between patches and the local information within each patch. In particular, we regarded the local patches (e.g., 16×16) as “visual sentences” and further divided them into smaller patches (e.g., 4×4) as “visual words.” A combination of the global feature information of “visual sentences” and the local feature information of “visual words” was used for modeling. In comparison to CNN and VT, MIM’s network is considered lightweight and less computationally intensive, while still being able to effectively extract both global and local information. The proposed model was applied to a private lung cancer histopathology dataset. The workflow of MIM is shown in **Figure 1**:

(a) We use the lung cancer histopathology dataset provided by the First Hospital of Jilin University as the training set, and the details of this dataset are described in Section2.
(b) During the data processing, we expanded the dataset by performing data enhancement operation, and then finally did image normalization.
(c) In this stage, the pre-processed data are input into MIM Layer, and the global information features and local information features in it are extracted using a multilayer perceptron.
(d) In this stage of classification, the output feature maps are classified using the standard classification head.
(e) Finally, our proposed MLP IN MLP is evaluated and the model is evaluated using four metrics: accuracy, precision, specificity, sensitivity and F1-score.

The main contributions of this paper are as follows. (1) A new deep learning based model (i.e., MIM) is proposed, which only uses multi-layer perceptron to do feature extraction for visual sentences and visual words respectively, which can extract local features and global features sufficiently to improve the overall classification performance. (2) A triple classification task was performed to identify the degree of lung adenocarcinoma infiltration, and a digital pathology image dataset for lung adenocarcinoma infiltration detection was prepared in collaboration with pathologists. The experimental results proved that the MIM model can effectively determine the degree of lung cancer infiltration and achieved a good performance of 95.31%. (3) The MIM model has good performance in detecting the degree of lung adenocarcinoma infiltration and shows good generalization performance in other cancer histopathology image classification tasks.

## 2 Materials and methods

### 2.1 Dataset

**Figure 2:**
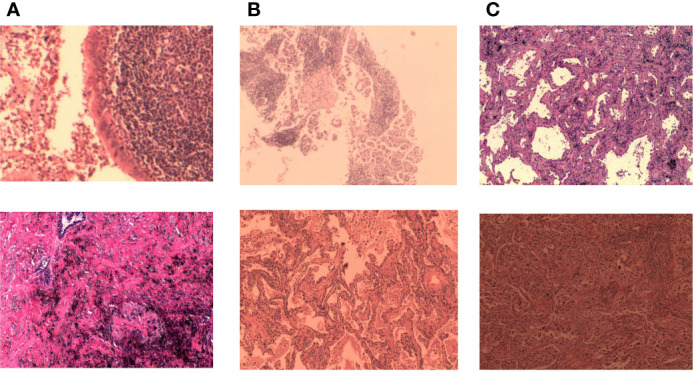
Some representative examples of lung adenocarcinoma data: **(A)** microinvasive lung adenocarcinoma, **(B)** invasive lung adenocarcinoma, and **(C)** images of normal lung tissue.

A unique dataset of lung cancer histopathological images provided by the First Hospital of Jilin University was used. The histopathological images were derived from 780 cases. Each case was given a clear diagnosis by a joint consultation of doctors from different departments, and the diagnosis was collated and labeled by four experienced doctors over a period of 3 months to ensure that each sample had considerable clinical features. Based on this approach, we cropped and cut the histopathological images and gave specific labels for each cut. The dataset includes two parts: training set and test set. Each part contains three categories (Normal, Infiltration, and Micro_infiltration). In the training set there are 7842 data, Infiltration and Micro_Infiltration include 2,614 and 2,614 digital pathology images respectively, and the remaining 2,614 pathology data are Normal. A total of 870 digital pathological images are included the test set. Among which, each category contains 290 lung pathological images. This dataset contains 8712 lung pathology images of size 2048×1500. Among the invasive and microinvasive lung adenocarcinomas, the tumors were predominantly of the adnexal type of growth, whereas microinvasive adenocarcinomas had infiltration foci of ≤0.5 cm in maximum diameter and were of the follicular, papillary, micropapillary, or solid type. Infiltrating adenocarcinomas had infiltration foci with a minimum diameter of >0.5 cm. Normal represented a benign area of lung tissue. Some samples of the specific data are shown in **Figure 2**.

### 2.2 Data preprocessing

Dataset pre-processing was performed, and images were subjected to resizing, data enhancement, and data normalization operations. All images were resized to 256×256 pixels. Five types of enhancement techniques (horizontal flip, rotation, scaling, height shift, and width shift) were then applied on the dataset, where the rotation was set to 15° and the height shift and width shift were set to 5% of the image size. These enhancement methods alter the relative position of cancer in histopathological images; therefore, the training set was expanded for training the MIM and no data enhancement operation was taken for the test set. The tumor tissues were extracted and sent to the pathology department; thus, each doctor may use different stains in the process of making tumor sections because of different personal habits. An image normalization operation was performed to turn the pathological images into black and white to avoid the color of the stain as a feature during model learning.

### 2.3 MLP-mixer

The MLP-Mixer does not use convolution operations or self-attentive mechanisms. Its architecture is based entirely on repeated application of multilayer perceptrons (MLPs) on spatial locations or feature channels, relying only on basic matrix multiplication routines, changes in data layout (reshaping and transposition), and scalar nonlinearities. Therefore, the model has a simple network and low computational effort. A simple MLP-based model that can compete with the best CNN and VT models available today was constructed.

**Figure 3:**
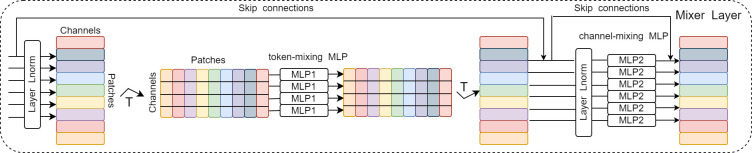
Detailed structure of Mixer Layer.

The overall structure of MLP-Mixer consists of three main parts as follows:

1. Per-patch Fully-connected: Linearly map the input image into a number of p×p×3-sized image patches, flatten each patch and linearly map it into a two-dimensional vector. The input image is thus converted into a two-dimensional (S,D) tensor, where S represents the number of patches and D represents the flattened dimensions of each patch. Specifically, $S=\frac{HW}{P2}$ (where H and W represent the height and width of the input image, respectively, and p represents the size of each patch), and D=3×p^2^ (where 3 represents the number of channels in the input image).
2. Mixer Layer: This layer consists of N identical layers. **Figure 3** shows the internal structure of the layers. Each layer consists of two types of MLPs: channel-mixing MLP and token-mixing MLP. Token-mixing MLP acts on the columns of a 2D table and learns the feature information between different spatial locations; it operates independently on each channel. Channel-mixing MLP acts on the rows of a 2D table and learns the feature information between different channels; it runs independently on the same spatial location. The output dimensions of both MLPs always remain the same as the input dimensions. The idea is to explicitly distinguish between per-position (channel-mixing) operations and cross-position (token-mixing) operations. In addition, other standard components are introduced, such as the residual structure (skip-connections) (19), which improves the ability of information interaction and avoids network degradation over deep gradients and the accuracy degradation of the training set. Layer normalization (20) is a key part of the model for stable training and faster convergence.
3. Finally, MLP-Mixer uses a standard classification head with the global average pooling layer followed by a linear classifier.

MLP-Mixer implements the convolutional and pooling layers of CNNs and the self-attentive layer of Transformer architecture using the two MLP types and demonstrates a simple MLP-based model that achieves competitiveness with current CNN or VT models, because MLPs require only simple mathematical operations.

### 2.4 Methods

What makes the MLP-Mixer model effective is that it can fully handle the relationship between several input patches to achieve global information extraction. However, this method also has an obvious drawback, that is, after the input image is split into several patches, it spreads each patch into a 1D vector and thus destroys the local information inside each patch. Therefore, we believe that MLP-Mixer modeling does not take into account the information inside each input patch.

By analyzing the lung histopathology dataset, we found that the histological features of lung cancer can be broadly classified as adnexal, alveolar, papillary, micropapillary, and solid (21). The cells that are likely to be presented in high magnification view are mostly tubular, columnar oval, and peg-shaped. Pathologists rely on information about these complex shape features to diagnose the type of cancer and the degree of infiltration.

**Figure 4:**
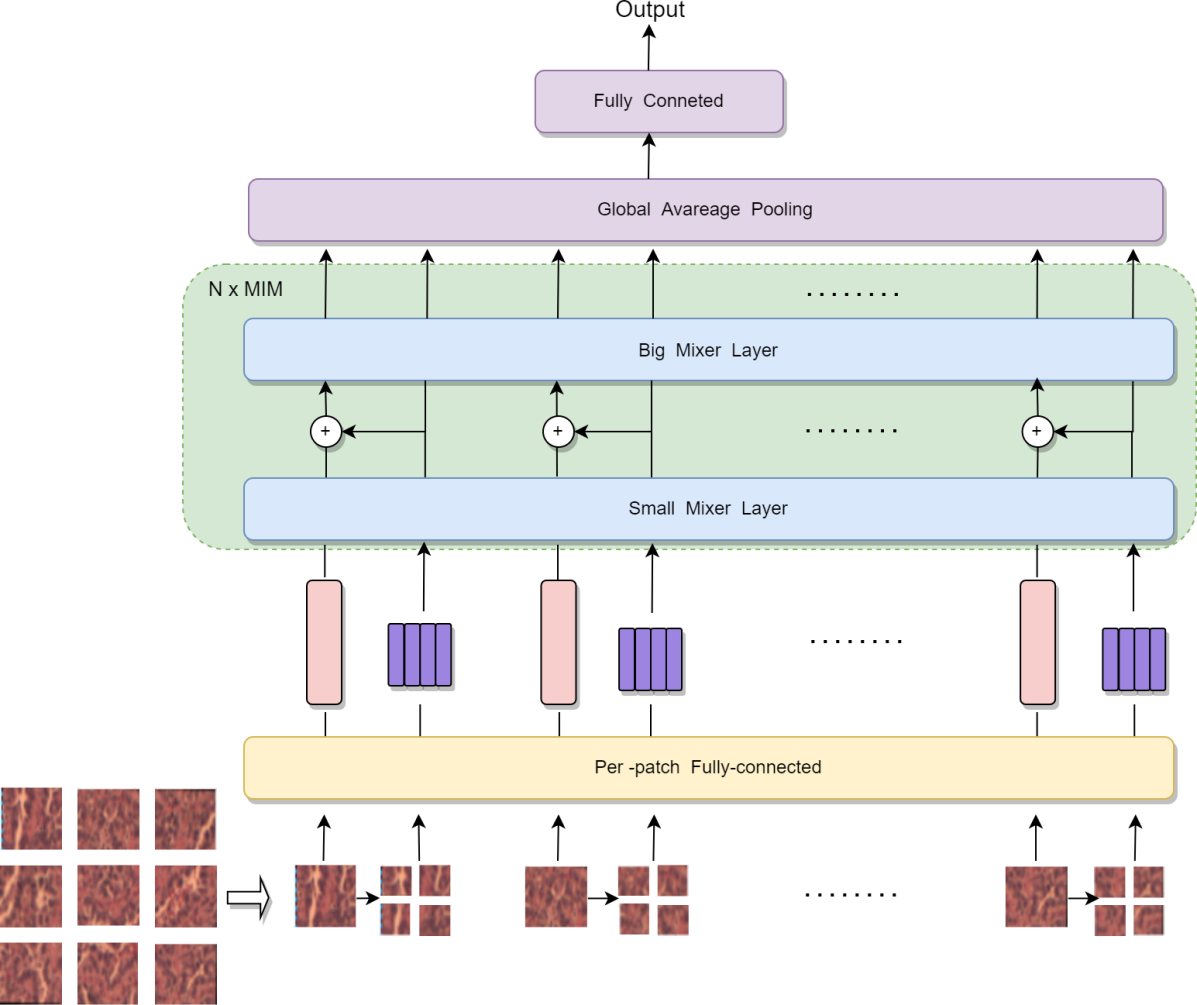
Internal structure of MLP IN MLP.

Digital pathological images are more highly complex than natural images with rich details and local information; therefore, the application of MLP-Mixer to medical pathology will be somewhat limited. It will fail to tap the cell and tissue features in different locations if the granularity of patch segmentation is not good enough. This paper proposes an MIM architecture to address the shortcomings of MLP-Mixer. The proposed model considers the global and local information between patches and the internal information of each patch, improving the performance of the model. The specific network structure is shown in **Figure 4**.

We partitioned the input 2D image into n patches uniformly and without overlapping. Let (p, p) be the resolution of each patch and X = [X_1_, X_2_, …, X_n_] ∈ R^n×p×p×3^. Each patch was spread into a 1D vector with a length of 3 × p². Finally, linear mapping was performed to obtain a 2D tensor table about patches (n, c) i.e. X∈R^n×c^. MLP-Mixer just designs a mixer layer to handle the relationship between n image patch sequences, unlike MIM, where two standard mixer layers extract the global and local information in the image. In MIM, we consider each patch as a visual sentence representing an image. Each patch will be further divided into m sub-patch visual words, that is, a visual sentence consists of a series of visual words: X_i_ → [X_i1_, X_i2_, …, X_im_] ∈ R^n×P×P×3^, where X_ij_ ∈ R^s×s×3^ is the j-th visual word of the i-th visual sentence, and (s, s) is the resolution size of the subpatch, j=1, 2, …, m. Each subpatch was also spanned into a 1D vector during the segmentation of the subpatch, and the 2D tensor table (n’, c’) about the subpatch was obtained after linear mapping. For example, the size of the visual sentence is 16×16×3. Here, p=16; therefore, the size of the visual word is 8×8×3. Each visual sentence was divided into four visual words, that is X_ij_ ∈ R^8×8×12^.

In MIM, we have two data flows in which one flow operates across the visual sentences, and the other processes the visual words inside each sentence. For the visual words, we utilize a small mixer layer as follows to explore the relation between visual words:


(1) $$Z_{ij}^{l}=\;X_{ij}^{l}\;+\;{\left(W_{2}\sigma (W_{1}{\left(LN(\;X_{ij}^{l})\right)}^{T})\right)}^{T}$$

(2) $$Y_{ij}^{l}=\;Z_{ij}^{l}\;+\;{\left(W_{4}\sigma (W_{3}{\left(LN(\;Z_{ij}^{l})\right)}^{T})\right)}^{T}$$

where l = 1, 2, …, L is the index of the l-th block, L is the total number of stacked blocks, T stands for transpose, σ is an element-wise nonlinearity (GELU) (22), W is a fully connected layer process, Z is the output after token-mixing MLP, and Y is the output after channel-mixing MLP. This process builds the relationship among visual words by computing the interactions between any two visual words. For example, in a patch of human face, a word corresponding to the eye is more related to other words of eyes and interacts less with forehead part. For the sentence level, the sequence of visual words are transformed into the domain of visual sentence by linear projection and added into the visual sentence as follows:


(3) $$U_{i}^{l}=X_{i}^{l}+FC(LN(Y_{ij}^{l}))$$

With the above addition operation, the representation of sentence sequences is augmented by word-level features. We used the standard mixer layer for transforming the visual sentences as follows:


(4) $$S_{\text{i}}^{\text{l}}=U_{i}^{l}\;+\;{\left(W_{2}\sigma (W_{1}{\left(LN(\;U_{i}^{l})\right)}^{T})\right)}^{T}$$

(5) $$Q_{i}^{l}=\;S_{i}^{l}\;+\;{\left(W_{4}\sigma (W_{3}{\left(LN(\;S_{i}^{l})\right)}^{T})\right)}^{T}$$

This big mixer layer is used for modeling the relationships among sentence sequences. In summary, the inputs and outputs of the MIM layer include the sequence of visual word and sequence of visual sentences as shown in **Figure 4**; hence, MIM can be formulated as:


(6) Y , Q=MIM(X , U)

In our MIM layer, the small mixer layer is used to model the relationship between visual words for local feature extraction, and the big mixer layer captures the intrinsic information from the sequence of visual sentences. We built the MIM network by stacking the MIM layer for N times. Finally, MIM uses a standard classification head with the global average pooling layer, followed by a linear classifier.

### 2.5 Hyperparameter settings

Finding the optimal values of hyperparameters is one of the crucial tasks for building a robust model. Besides fully extracting image information, the hyperparameters values have a great influence on the fast convergence of the model. MIM was used to train the image of the lung cancer pathological tissue dataset for 200 epochs. In each epoch, batch size is set to 256. It uses a method to train the lung adenocarcinoma infiltration level recognition task from scratch. AdamW (23) was used as the optimizer, and its parameters were set as 3×10−5 learning rate. In addition, the dropout ratio (24) was set to 0.1 in the training process.

### 2.6 Experimental environment

The experiments were carried out on a local workstation with 32 GB RAM and Windows 10 operating system. The GPU of the workstation consists of NVIDIA RTX 3090 and CPU Intel Core i9-10875H 2.30 GHz. We used the Python 3.7 programming language and PyTorch 1.8.0.

### 2.7 Performance metrics

Accuracy, precision, sensitivity, specificity and F1-score were calculated to evaluate the performance of MIM. Here, accuracy indicates the proportion of the number of samples correctly predicted by the model to the total number of samples. Precision indicates the proportion of samples correctly predicted by the model as a percentage of all samples predicted by the model to be positive. Sensitivity indicates the proportion of the number of samples correctly predicted by the model as a percentage of the total number of positive samples; the specific value indicates the proportion of the number of samples correctly predicted by the model as a percentage of the total number of negative samples. F1-score combines the results of precision and sensitivity. True positive, true negative, false positive, and false negative were used in the definition of these five criteria in **Table 1**.

**Table 1:** Criteria and corresponding definitions for image global detection evaluation.

Criterion	Definition	Criterion	Definition
Accuracy	$\frac{TP+TN}{TP+TN+FP+FN}$	Precision	$\frac{TP}{TP+FP}$
Sensitivity	$\;\frac{TP}{TP+FN}$	Specificity	$\frac{TN}{\;TN+FP}$
F1-score	$\frac{2\times \text{Precision}\times \text{Sensitivity}}{\text{Precision}+\text{Sensitivity}}$		

## 3 Result

### 3.1 Experimental results and analysis

In the test phases, accuracy, precision, sensitivity, specificity and F1-score were calculated to evaluate the performance of the proposed MIM and other networks. The results are shown in **Table 2**. The accuracy, precision, sensitivity, specificity and F1-score of MIM on the test set were 95.31%, 93.09%, 93.10%, 96.43% and 93.10%, respectively. The accuracy, precision, sensitivity, specificity and F1-score of MLP-Mixer on the test set were 92.43%, 88.97%, 88.96%, 94.24% and 88.97%, respectively. The five evaluation metrics of the proposed MIM were on average nearly 3% higher than those of MLP-Mixer. In addition, MIM had higher evaluation metrics than ResNet50 and Swin Transformer (25). The effectiveness of modeling using a combination of global and local information in the MIM model was demonstrated for the large amount of local information present in lung adenocarcinoma.

**Table 2:** Results of MLP IN MLP and other networks on private datasets (In %).

Model	Accuracy	Precision	Sensitivity	Specificity	F1-score
MLP IN MLP	95.31	93.09	93.10	96.43	93.10
MLP-Mixer (18)	92.43	88.97	88.96	94.24	88.97
ResNet50 (19)	93.95	91.46	91.14	95.36	91.30
Swin (25)	94.60	92.48	92.06	95.85	92.27

**Figure 5:**
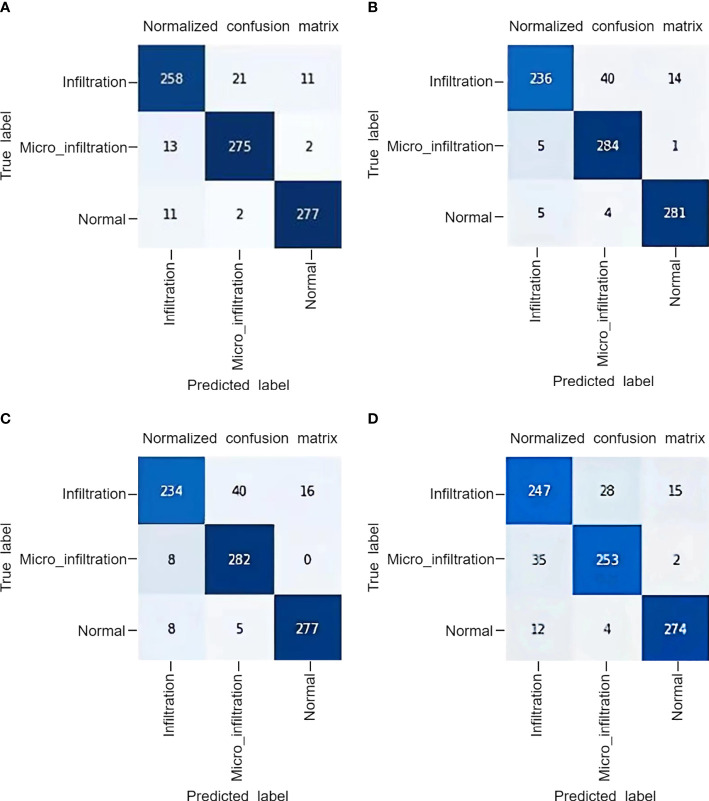
The confusion matrix obtained by the model on a private dataset: **(A)** MLP IN MLP, **(B)** Swin Transformer, **(C)** ResNet50, **(D)** MLP-Mixer.

The superior performance of MIM can be further demonstrated by the correlation model confusion matrix shown in **Figure 5**. In the test confusion matrix, the classifications of normal lung pathological data are very accurate. The classification errors of ResNet and Swin Transformer are shown in **Figures 5B** and **C**, respectively. Both methods misclassified Infiltration as Micro_infiltration. In **Figure 5D**, the misclassification of MLP-Mixer is due to the confusion caused between Infiltration and Micro_infiltration. The main reason for these phenomena are that the Infiltration and Micro_infiltration samples contain tumors, but the only difference is whether the infiltrated area is more than 5 mm. Moreover, the model gave wrong judgments for some samples near the threshold. As shown in **Figure 5A**, MIM effectively alleviated these problems. Using MIM, only 21 Infitration data were incorrectly predicted as Micro_infiltration data, and 13 Micro_Infitration data were incorrectly predicted as Infitration data. The effectiveness of the MIM modeling approach using local and global information was further demonstrated in terms of the degree of infiltration of lung adenocarcinoma, allowing the effective identification of differences in infiltrative and microinfiltrative pathological features.

### 3.2 Extended experiments

#### 3.2.1 Extended experiment on combined dataset: private and public data on stomach cancer

**Figure 6:**
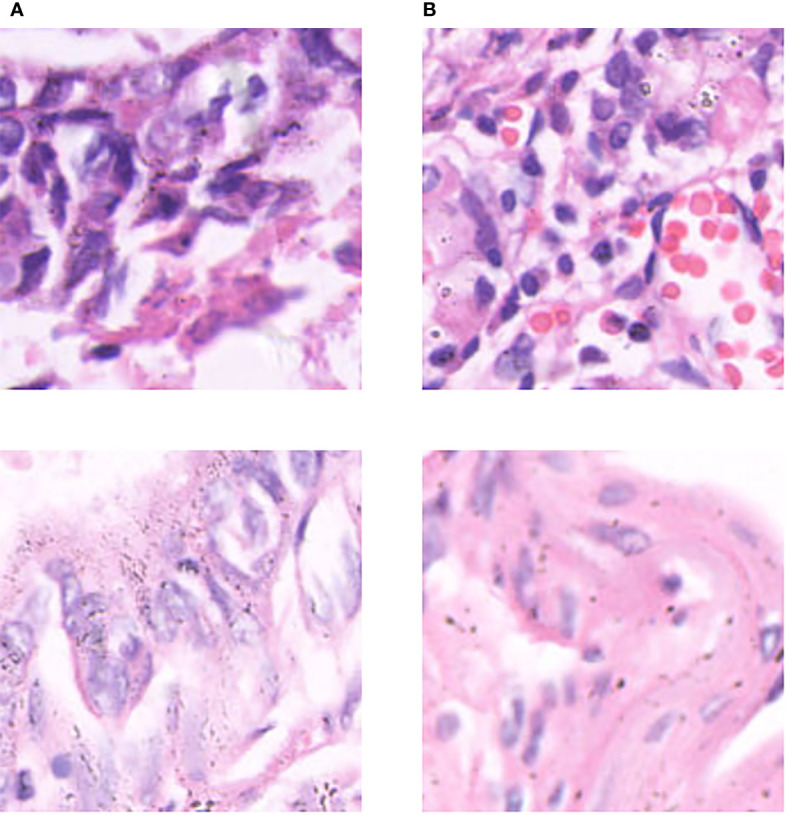
Some examples in the gastric cancer dataset: **(A)** normal stomach tissue data, **(B) **abnormal stomach tissue data.

A hybrid dataset consisting of the publicly available pathological dataset of gastric cancer from Northeastern University (26) and our private dataset was used to come up with extended experiments to explore the performance of the model on lung tissue and other histopathological data. The experimental setup was the same as the main experimental setup. The gastric histopathology database from Northeastern University has three sizes (160×160, 120×120, and 80×80 pixels), and two data types (normal and abnormal). Some examples are shown in **Figure 6**. In this experiment, 160×160 size data were used and resized to the same size (256×256) as our private dataset during data processing. We use 10080 normal data images and 6670 abnormal data images. Considering the number of training and test sets in our private dataset, we randomly partitioned the training and test sets in a 9:1 ratio for the histopathological data of gastric cancer. The mixed data set is a five-category task. The combined dataset’s data settings are shown in **Table 3**.

**Table 3:** Data setting for training and test sets.

Class/Dataset	Train	Test	Sum
Micro_infiltration	2614	290	2904
Infiltration	2614	290	2904
Normal	2614	290	2904
Stomach_Abnormal	6013	657	6670
Stomach_Normal	9072	1008	10080
Sum	22927	2535	25462

Six representative models were selected for the comparative experiments with MIM, including two CNN models [VGG16 (27) and ResNet50] two VT models [ViT and XCiT (28)], and two MLP models [ResMLP (29) and MLP-Mixer]. The experimental settings were the same as the previoussetting. **Table 4** shows the results of the models on the test set of the combined dataset. ViT obtained the highest accuracy rate of 95.50% among the six models, and MLP-Mixer had the lowest accuracy rate of 91.45%. Among the models, the proposed MIM model achieved the highest test accuracy of 96.50%, which was nearly 1% higher than the accuracy of ViT and nearly 5% more accurate than MLP-Mixer. In addition, the precision, sensitivity, specificity and F1-score of MIM were also the highest at 93.95%, 93.89%, 98.91% and 93.92%, respectively. The results show the effectiveness of the proposed MIM model.

**Table 4:** Comparison results of models on the test set of the combined dataset (In %).

Model	Accuracy	Precision	Sensitivity	Specificity	F1-score
MLP IN MLP	96.50	93.95	93.89	98.91	93.92
ResNet50 (19) Vgg16 (27) XCiT (28) ViT (16) ResMLP (29) MLP-Mixer (18)	94.90 93.68 95.05 95.50 94.87 91.45	92.44 93.70 87.40 92.49 92.43 87.12	92.34 93.53 89.66 92.37 92.10 87.19	98.46 98.12 97.85 98.53 98.39 97.62	92.39 93.62 88.53 92.43 92.27 87.16

#### 3.2.2 Extended experiment on combined dataset: private data and public data

**Figure 7:**
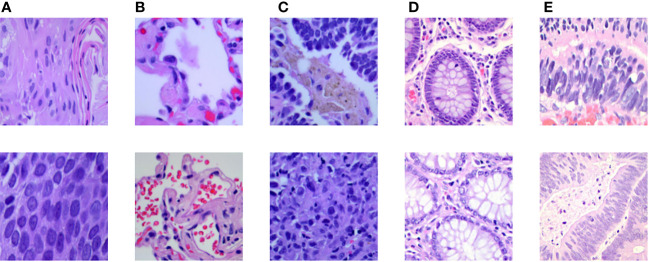
Some examples in the LC25000 dataset: **(A)** lung squamous carcinoma histopathology picture, **(B)** normal lung histopathology image, **(C)** lung adenocarcinoma histopathology image, **(D)** normal colon histopathology image, **(E)** colon adenocarcinoma histopathology image.

In addition to exploring the sensitivity between different histopathologies, we also incorporated other lung cancer subtypes to further increase the complexity of the experiment. The public dataset, LC25000 (30), was incorporated to the private dataset, and extended experiments were performed on the hybrid dataset to validate the generalization ability of MIM on other lung cancer subtype pathology datasets. The experimental setup was the same as the main experimental setup. The LC25000 dataset has five different data types about lung and colon histopathology: normal lung histopathology image (Lung_n), lung squamous carcinoma histopathology image (Lung_scc), lung adenocarcinoma histopathology image (Lung_aca), normal colon histopathology image (Colon_n), and colon adenocarcinoma histopathology image (Colon_aca). Some examples are shown in **Figure 7**. The dataset has 4500 images in the training set and 500 images in the test set for each type, for a total of 25000 images for the five data types. Both datasets contain normal lung histopathology data; hence, we set all normal lung tissue data as one category, forming a seven-category hybrid dataset. **Table 5** shows the data settings.

**Table 5:** Data setting for training and test sets.

Class/Dataset	Train	Test	Sum
Micro_infiltration	2614	290	2904
Infiltration	2614	290	2904
Lung_aca	4500	500	5000
Lung_scc	4500	500	5000
Lung_n	7114	790	7904
Colon_aca	4500	500	5000
Colon_n	4500	500	5000
Sum	30342	3370	33712

The results of the comparison tests between MIM and six classical deep learning models in the lung cancer and colon cancer detection task are shown in **Table 6**. Obviously, among the six classical models, ConViT (33) performed the best in the test set with accuracy, precision, sensitivity, specificity and F1-score values of 97.83%, 96.70%, 96.43%, 99.59% and 96.52%, respectively. The accuracy, precision, sensitivity, specificity and F1-score values of MIM on the test set were 98.09%, 97.17%, 97.04%, 99.67% and 97.11%, respectively. The specific detection results of MIM are shown in **Table 7**. For the five types of lung pathological images, 2309 images were correctly detected by the MIM model, and only 61 images were not detected; therefore, the accuracy of MIM in this set was 97.42%. For colon adenocarcinoma histopathological images, 996 images were detected by the MIM model, and only four images were not detected; therefore, the accuracy of the model for colorectal images was 99.60%. The LC2500 dataset contains lung cancer subtypes different from invasive lung cancer in addition to the colon histopathological data that are also present. The findings indicate that MIM has good classification performance between different lung cancer subtypes and performs well for colon histopathological data.

**Table 6:** A comparison of image classification results on the mixed test set.

Model	Accuracy	Precision	Sensitivity	Specificity	F1-score
MLP IN MLP	98.09	97.17	97.04	99.67	97.11
EfficientNet (31)	96.96	95.22	95.45	99.32	95.34
InceptionV2 (32)	96.76	95.29	95.31	99.43	95.30
Swin (24)	97.51	96.09	95.97	99.53	96.03
ConViT (33)	97.83	96.70	96.43	99.59	96.52
gMLP (34)	96.41	96.09	95.16	99.35	95.63
MLP Mixer (17)	95.54	92.71	92.07	99.00	92.39

(In %).

**Table 7:** The result of MLP IN MLP in classification task.

Data Type	Correct	Incorrect	Accuracy
Lung histopathology image	2039	61	97.42
Colon histopathology image	996	4	99.60
Sum	3305	65	98.09

(In %).

## 4 Discussion

First, in our experiments, we found that the performance is better when the number of MIM blocks is set to 8, when the parameter size of MIM is about 165 MB. We had 7842 training data and used 200 epochs to train MIM, and the total time was about 230 minutes. We lightened the model according to the actual needs of clinical work (35). First, we used a quantization technique to reduce the number of bits required to store each weight from 32 bits to 16 bits. Then, the size of the model parameters was reduced to 83 MB. Consequently, the classification performance was almost unchanged, and the total training time was reduced by about 55 minutes.

Second, the segmentation granularity of patches varies in terms of the feature richness that can be extracted from the image. By contrast, the feature extraction layer of MIM has two different data streams, namely, visual sentences and visual words, and MIM was designed to capture the global feature information between patches and mine the local feature information within patches. In the pre-experiments, we set different resolutions for the visual sentences and visual words. For the fairness of the experiments, the number of feature extraction layers in the experiments was set to 8. The rest of the experimental settings and the datasets used were the same as those in the main experiments. The specific experimental details and results are shown in **Table 8**. The highest accuracy rate of 95.31% was achieved with a visual sentence resolution of 16 and a visual word resolution of 4. The finer the granularity of patch segmentation, the more the number of patches. Additionally, the amount of model computation was positively correlated with the number of patches. The fullest possible exploitation of the internal local information of patches should also be considered. Therefore, the resolutions of vision sentences and vision words were set to 16×16 and 4×4, respectively, considering the computational power and training time of the model.

**Table 8:** Comparison test of different patch sizes.

Model	Visual Sentences	Visual word	Accuracy
MIM	32	8	93.24
	32	4	94.12
	16	8	94.78
	16	4	95.31

(In %).

Third, the optimizer and activation function should be chosen carefully. Although various optimizers (e.g., stochastic gradient descent (36) and Lagrangian optimizer) and activation functions (e.g., CroReLU) (37, 38) are implemented with deep networks to solve different classification problems, we applied AdamW and GELU because of their efficiency in the proposed architecture with our datasets. AdamW solves the problem of parameter over-fitting with Adam optimizer (39) by introducing the L2 regularization terms of parameters in the loss function. It is the fastest optimizer for gradient descent speed and training neural networks that is used in all models. GELU has been widely used in the recent Transformer model. Randomness is introduced to make the model training process more robust and avoid the problem of gradient disappearance well. Loss functions have an important role in a deep network architecture. Hybrid loss functions have been proposed in several works to improve the performance of model. We used the default loss function [i.e., cross-entropy (40)] because of its low computational cost and efficiency with our pathological images.

Finally, although the proposed model showed a prominent performance in the classifying degree of lung cancer infiltration, this study encountered some challenges. Our work was limited to two types of lung cancer data, namely, infiltration and microinfiltration, because of data inadequacy. Moreover, we only considered digital pathological images. Other medical imaging techniques, such as computed tomography and magnetic resonance imaging, can also be taken under consideration in future work. In addition, this work can be extended to patients’ medical history (age, gender, physical condition, etc.) with image datasets for more accurate predictions.

## 5 Conclusion

In the experiments, MIM was tested on a lung cancer pathology dataset with 95.31% accuracy, showing the model’s potential in lung cancer determination tasks. The lung pathological images have a large amount of information on histological shapes, such as pegs and papillae. The local structure of patches was destroyed in the MLP-Mixer model. Compared with the conventional MLP-Mixer, MIM can better preserve and model local information for lung histopathology identification tasks. In the extended experiments, MIM is more efficient and performs better than CNN, MLP and VT models proving its powerful generalization ability.

## Data availability statement

The original contributions presented in the study are included in the article/supplementary material. Further inquiries can be directed to the corresponding authors.

## Ethics statement

Written informed consent was obtained from the individual(s) for the publication of any potentially identifiable images or data included in this article.

## Author contributions

The conception or design of the work: ML, LYL, and TZ; the acquisition, analysis, or interpretation of data: YGL, FW, TZ, and YPL; drafting the work or revising: YS, XG, and HW; provide approval for publication of the content: NS, JZ, and KS; agree to be accountable for all aspects of the work: ML and LL. All authors contributed to the article and approved the submitted version.

## References

[1] McIntyre A Ganti AK. Lung cancer–a global perspective. J Surg Oncol (2017) 115(5):550–4. doi: 10.1002/jso.24532 28418583

[2] Myers DJ Wallen JM. Lung adenocarcinoma. StatPearls. StatPearls Publishing (2021). 30137862

[3] Donington JS. Progress in the management of early stage non-small cell lung cancer in 2017. J Thorac Oncol (2018) 13.6:767–78. doi: 10.1016/j.jtho.2018.04.002 29654928

[4] Travis WD. Pathology of lung cancer. Clinics Chest Med (2011) 32 (4):669–92. doi: 10.1016/S0272-5231(03)00061-3 22054879

[5] Ruffini E Rena O Oliaro A. Lung tumors with mixed histologic pattern. clinico-pathologic characteristics and prognostic significance. Eur J cardio-thoracic Surg (2002) 22(5):701–7. doi: 10.1016/S1010-7940(02)00481-5 12414033

[6] Le Cun Y Bengio Y Hinton G. Deep learning. Nature (2015) 521(7553):436–44. doi: 10.1038/nature14539 26017442

[7] Shen D Wu G Suk HI. Deep learning in medical image analysis. Annu Rev Biomed Eng (2017) 19:221. doi: 10.1146/annurev-bioeng-071516-044442 28301734 PMC5479722

[8] Hua KL Hsu CH Hidayati SC. Computer-aided classification of lung nodules on computed tomography images *via* deep learning technique. OncoTargets Ther (2015) 8:2015–22. doi: 10.2147/OTT.S80733 PMC4531007 26346558

[9] Roy TS Sirohi N Patle A. Classification of lung image and nodule detection using fuzzy inference system, in: International conference on computing, communication & automation IEEE (2015), 1204–7.

[10] Coudray N Ocampo PS Sakellaropoulos T. Classification and mutation prediction from non–small cell lung cancer histopathology images using deep learning. Nat Med (2018) 24(10):1559–67. doi: 10.1038/s41591-018-0177-5 PMC9847512 30224757

[11] Wang X Chen H Gan C. Weakly supervised deep learning for whole slide lung cancer image analysis. IEEE Trans cybernetics (2019) 50(9):3950–62. doi: 10.1109/TCYB.2019.2935141 31484154

[12] Shen W Zhou M Yang F. Multi-crop convolutional neural networks for lung nodule malignancy suspiciousness classification. Pattern Recognition (2017) 61:663–73. doi: 10.1016/j.patcog.2016.05.029

[13] Kriegsmann M Haag C Weis CA. Deep learning for the classification of small-cell and non-small-cell lung cancer. Cancers (2020) 12(6):1604. doi: 10.3390/cancers12061604 32560475 PMC7352768

[14] Khademi S Heidarian S Afshar P. Spatio-Temporal Hybrid Fusion of CAE and SWin Transformers for Lung Cancer Malignancy Prediction[C]//ICASSP 2023-2023 IEEE International Conference on Acoustics, Speech and Signal Processing (ICASSP). IEEE (2023) 2023:1–5. doi: 10.1109/ICASSP49357.2023.10094986

[15] O'Shea K Nash R. An introduction to convolutional neural networks[J]. arXiv preprint arXiv:1511.08458 (2015).

[16] Dosovitskiy A Beyer L Kolesnikov A. An image is worth 16x16 words: transformers for image recognition at scale. arXiv (2020) 20. doi: 10.48550/arXiv.2010.11929

[17] Dani S Hanwate PS Panse H. Survey on the use of CNN andDeep Learning in Image Classification[J]. J. Emerg. Technol. Innov. Res (2021) 8:609–611.

[18] Tolstikhin IO Houlsby N Kolesnikov A. Mlp-mixer: an all-mlp architecture for vision. Adv Neural Inf Process Syst (2021) 34:24261–72.

[19] He K Zhang X Ren S. Deep residual learning for image recognition, in: Proceedings of the IEEE conference on computer vision and pattern recognition (2016), 770–8.

[20] Ba JL Kiros JR Hinton GE. Layer normalization. arXiv preprint arXiv:1607.06450 (2016). doi: 10.48550/arXiv.1607.06450

[21] Terasaki H Niki T Matsuno Y. Lung adenocarcinoma with mixed bronchioloalveolar and invasive components: clinicopathological features, subclassification by extent of invasive foci, and immunohistochemical characterization. Am J Surg Pathol (2003) 27(7):937–51. doi: 10.1097/00000478-200307000-00009 12826886

[22] Hendrycks D Gimpel K. Gaussian Error linear units (gelus). arXiv preprint arXiv:1606.08415 (2016). doi: 10.48550/arXiv.1606.08415

[23] Loshchilov I Hutter F. Decoupled weight decay regularization. arXiv preprint arXiv:1711.05101, (2017). doi: 10.48550/arXiv.1711.05101

[24] Srivastava N Hinton G Krizhevsky A. Dropout: a simple way to prevent neural networks from overfitting. J Mach Learn Res (2014) 15(1):1929–58. doi: 10.1145/3340555.3353730

[25] Liu Z Lin Y Cao Y. (2021). Swin transformer: hierarchical vision transformer using shifted windows, in: Proceedings of the IEEE/CVF International Conference on Computer Vision, . pp. 10012–22.

[26] Hu W Li C Li X. GasHisSDB: a new gastric histopathology image dataset for computer aided diagnosis of gastric cancer. Comput Biol Med (2022) 142:105207. doi: 10.1016/j.compbiomed.2021.105207 35016101

[27] Simonyan K Zisserman A. Very deep convolutional networks forlarge-scale image recognition[J]. arXiv preprint arXiv:1409.1556 (2014). doi: 10.48550/arXiv.1409.1556

[28] Ali A Touvron H Caron M. Xcit: cross-covariance image transformers. Adv Neural Inf Process Syst (2021) 34:20014–27. doi: 10.48550/arXiv.2106.09681

[29] Touvron H Bojanowski P Caron M. Resmlp: feedforward networks for image classification with data-efficient training. IEEE Trans Pattern Anal Mach (2022) 45:5314–5321. doi: 10.1109/TPAMI.2022.3206148 36094972

[30] Borkowski AA Bui MM Thomas LB. Lung and colon cancer histopathological image dataset (lc25000)[J]. arXiv preprint (2019) arXiv:1912.12142. doi: 10.48550/arXiv.1912.12142

[31] Tan M Le Q. (2019). Efficientnet: rethinking model scaling for convolutional neural networks, in: International conference on machine learning, . pp. 6105–14. PMLR.

[32] Szegedy C Vanhoucke V Ioffe S. (2016). Rethinking the inception architecture for computer vision, in: Proceedings of the IEEE conference on computer vision and pattern recognition, . pp. 2818–26.

[33] d’Ascoli S Touvron H Leavitt ML. (2021). Convit: improving vision transformers with soft convolutional inductive biases, in: International Conference on Machine Learning, . pp. 2286–96. PMLR.

[34] Liu H Dai Z So D. Pay attention to mlps. Adv Neural Inf Process Syst (2021) 34:9204–15. doi: 10.48550/arXiv.2105.08050

[35] Choudhary T Mishra V Goswami A. A comprehensive survey on model compression and acceleration. Artif Intell Rev (2020) 53(7):5113–55. doi: 10.1007/s10462-020-09816-7

[36] Bottou L. Stochastic gradient descent tricks. In: Neural networks: tricks of the trade. Berlin, Heidelberg: Springer (2012). p. 421–36.

[37] Goceri E. (2019). Analysis of deep networks with residual blocks and different activation functions: classification of skin diseases, in: 2019 Ninth international conference on image processing theory, tools and applications (IPTA), . pp. 1–6. IEEE.

[38] Liu Y Wang H Song K. CroReLU: cross-crossing space-based visual activation function for lung cancer pathology image recognition. Cancers (2022) 14(21):5181. doi: 10.3390/cancers14215181 36358598 PMC9657127

[39] Kingma DP Ba J. Adam: A method for stochastic optimization. arXiv (2014). doi: 10.48550/arXiv.1412.6980

[40] De, Boer PT Kroese DP Mannor S. A tutorial on the cross-entropy method. Ann operations Res (2005) 134(1):19–67. doi: 10.1007/s10479-005-5724-z

